# Unique hypersensitivity syndrome secondary to selpercatinib

**DOI:** 10.1016/j.jdcr.2024.06.008

**Published:** 2024-06-18

**Authors:** Siddharth Srikakolapu, Dev R. Sahni, Lauren M. Madigan

**Affiliations:** a Department of Medicine, Brookwood Baptist Health, Birmingham, Alabama; b Department of Dermatology, Brigham and Women&apos;s Hospital, Boston, Massachusetts; c Department of Dermatology, University of Utah, Salt Lake City, Utah

**Keywords:** drug-induced hypersensitivity syndrome, drug reaction with eosinophilia and systemic symptoms, hypersensitivity, oncodermatology, selpercatinib

## Introduction

Selpercatinib is a novel agent which received accelerated approval in 2020 for the treatment of RET-mutant malignancies including nonsmall cell lung cancer, medullary thyroid cancer, and other RET-fusion positive thyroid malignancies.^1^ As an oral RET kinase inhibitor, selpercatinib disrupts oncogenesis by suppressing tumor cell proliferation.^2^ Reported adverse reactions to therapy include hemorrhage, hepatotoxicity, impaired wound healing, hypertension, and QT prolongation.^1^ Cutaneous adverse events have also been described with treatment-related rashes of all grades developing in 20% and 14.5% of patients with RET-fusion positive nonsmall cell lung cancer and thyroid cancer respectively within clinical trials—though only few with high grade events.^3^^,^^4^ More recently, cutaneous eruptions occurring in the setting of systemic hypersensitivity have been further described.^5^ We report a case of a patient experiencing a hypersensitivity reaction with both cutaneous and systemic manifestations associated with selpercatinib administration and review what is currently known regarding this syndrome and its management.

## Case report

An elderly male in his 70s with a history of RET-fusion positive medullary thyroid cancer with known liver metastases presented to the emergency department with worsening malaise, high fevers, hypotension, and a cutaneous eruption of 2 days duration. The rash was mildly pruritic and initially presented on his face—with associated facial swelling—with subsequent cephalocaudal progression. The patient denied mucosal symptoms. His recent history was notable for initiation of 160 mg of oral selpercatinib twice daily for 6 days prior to presentation. He denied previous treatment with an immune checkpoint inhibitor.

**Fig 1:**
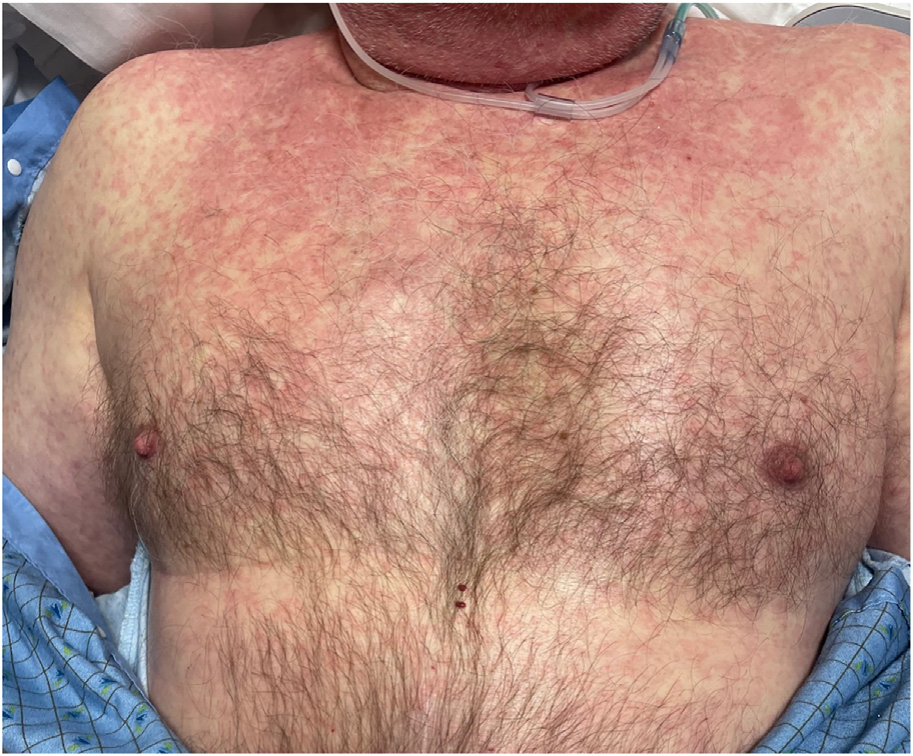
Symmetrically distributed erythematous macules and papules coalescing into patches and plaques on the chest, abdomen, and arms.

**Fig 2:**
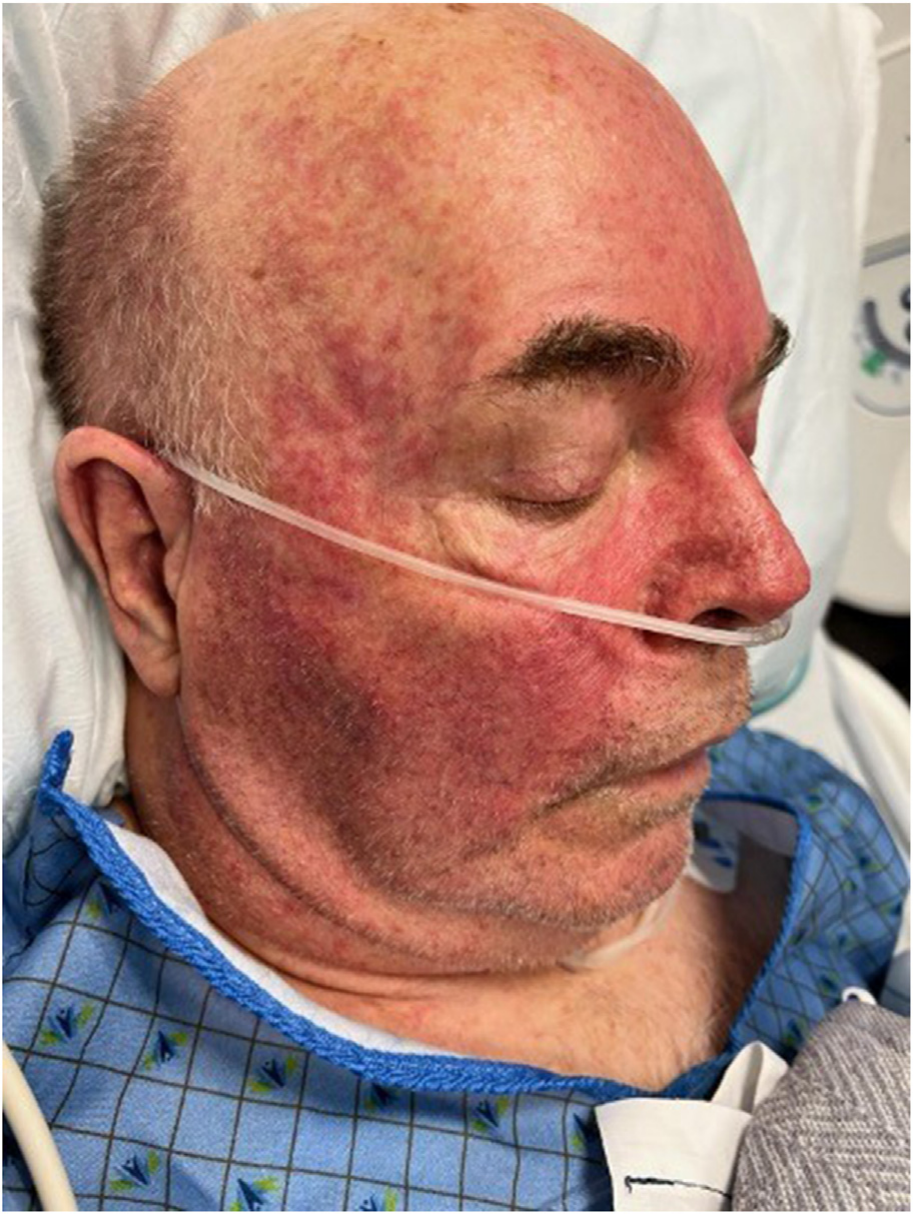
Periorbital and facial edema with erythematous patches and plaques on the forehead, nose, and cheeks.

There was initial concern for sepsis and he was placed on broad spectrum antibiotics while awaiting culture results. Dermatology was consulted to assist with management given the severity of his rash. Physical examination revealed an ill-appearing man with a diffuse morbilliform eruption involving the face, neck, trunk, bilateral arms, and proximal legs (Fig 1). Significant facial erythema and edema were also present (Fig 2). No lymphadenopathy or ocular, oral, or genital mucositis were appreciated. The patient was febrile (106.3 °F), tachycardic, and normotensive. Laboratory evaluation revealed elevations in serum creatinine (1.76 mg/dL), as well as mild elevations in alanine transaminase (70 u/L) and aspartate transferase (72 u/L). Thrombocytopenia (102 μ/L) without leukocytosis or eosinophilia was also noted. Skin biopsy was deferred due to high clinical suspicion for drug-induced hypersensitivity. The patient was subsequently started on 1 mg/kg of prednisone with rapid improvement in erythema, fevers, and facial swelling within 48 hours and antibiotics were discontinued. He completed a short steroid taper and patient was allowed to recover for approximately 1 week. The patient was then started on selpercatinib 40 mg twice daily. Within 2 hours of the first dose, the patient again developed fevers, rashes, and facial swelling and presented for admission. High dose steroids at 1 mg/kg of prednisone were started with rapid resolution of symptoms and patient was discharged the following day on a short steroid taper. Two weeks later, patient was imaged with an abdominal magnetic resonance imaging, and the primary lesion was decreased in size and other enhancing liver foci were not visualized. He subsequently started on cabozantinib 60 mg and remains on drug with stable disease.

## Discussion

In a recent review of data from the selpercatinib phase 1/2 LIBRETTO-001 trial, systemic hypersensitivity was noted to develop in approximately 7% of patients.^5^ This hypersensitivity reaction was defined by the presence of a maculopapular rash, typically preceded by fever and associated arthralgias/myalgias, with the addition of one or more of the following signs/symptoms: thrombocytopenia, elevated creatinine, elevated aspartate aminotransferase or alanine aminotransferase, hypotension, or tachycardia.^5^ In the LIBRETTO-001 analysis, the association of prior immune checkpoint inhibitor therapy was specifically addressed and, as hypothesized, those with prior exposure were more likely to experience systemic hypersensitivity—similar to what is observed with other kinase inhibitors.^5^

Notably, patients with selpercatinib-associated hypersensitivity appeared morphologically similar to patients with drug-induced hypersensitivity syndrome (also known as drug reaction with eosinophilia and systemic symptoms), though with a few unique considerations. These patients typically presented with symptoms after a delay of approximately 1.7 weeks of therapy and tended to lack lymphadenopathy and peripheral eosinophilia. Additionally, these reactions tended to resolve more rapidly (an average of 8 days compared to a protracted course over several weeks which may occur with drug-induced hypersensitivity syndrome).

Management of hypersensitivity reactions secondary to selpercatinib also differs, most notably with regard to medication re-exposure. Current recommended treatment includes initiation of systemic corticosteroids (oral prednisone) at a dose of 1 mg/kg daily, or equivalent, with appropriate prophylaxis. This therapy is then continued until symptom resolution, at which point selpercatinib can be restarted at a reduced dose 3 “dose levels” below the dose which preceded the onset of hypersensitivity (“dose levels” defined as 120 mg twice daily (level 1), 80 mg twice daily (level 2), and 40 mg twice daily (level 3)).^5^ The dose may then be increased by one level each week, as tolerated, until a target dose level is achieved. At that time, prednisone therapy is tapered. If hypersensitivity reoccurs, the above protocol may be repeated—or if the recurrence is very mild—selpercatinib may be continued uninterrupted with symptom-directed care. Ultimately, if a dose of even 40 mg twice daily is not tolerated, selpercatinib should be permanently discontinued.^5^

It is important for oncologists and dermatologists to be familiar with the unique hypersensitivity syndrome associated with selpercatinib. Recognition of this distinct reaction is important to ensure correct treatment including consideration of medication reintroduction when appropriate. With early identification, therapy can be quickly instituted and an important treatment option may be potentially salvaged.

## Conflicts of interest

None disclosed.
